# Randomised trial of once- or twice-daily MMX mesalazine for maintenance of remission in ulcerative colitis

**DOI:** 10.1136/gut.2007.138248

**Published:** 2008-02-13

**Authors:** M A Kamm, G R Lichtenstein, W J Sandborn, S Schreiber, K Lees, K Barrett, R Joseph

**Affiliations:** 1 Department of Medicine, St Vincent’s Hospital, Melbourne, Australia; 2 Division of Gastroenterology, University of Pennsylvania, Philadelphia, USA; 3 Inflammatory Bowel Disease Clinic, Mayo Clinic, Rochester, Minnesota, USA; 4 First Department of Medicine, Christian-Albrechts-Universität, Kiel, Germany; 5 Shire Pharmaceuticals Inc., Wayne, Philadelphia, USA; 6 Shire Pharmaceuticals Inc., Basingstoke, Hampshire, UK

## Abstract

**Aim:**

Maintenance treatment in ulcerative colitis should be as convenient as possible, to increase the chance of compliance. MMX mesalazine is a once-daily, high-strength (1.2 g/tablet) formulation of 5-aminosalicylic acid. This study evaluated the safety and efficacy of MMX mesalazine dosed once or twice daily as maintenance therapy in patients with ulcerative colitis.

**Methods:**

This multicentre, randomised, open-label trial enrolled patients with strictly defined clinical and endoscopic remission, immediately following an episode of mild to moderate ulcerative colitis. Patients were randomised to MMX mesalazine 2.4 g/day as a single (2×1.2 g tablet) or divided dose (1×1.2 g tablet twice daily) for 12 months.

**Results:**

174 patients (37.9%; safety population n = 459) experienced 384 adverse events, the majority of which were mild or moderate in intensity. Eighteen patients (3.9%), nine in each group, experienced a total of 22 serious adverse events (10 in the once-daily and 12 in the twice-daily group). Most serious adverse events were gastrointestinal, experienced by 5 patients in the once-daily and 4 in the twice-daily group. At month 12, 64.4% (efficacy population, n = 451) of patients in the once-daily and 68.5% of patients in the twice-daily group were in clinical and endoscopic remission (p = 0.351). At month 12, 88.9% and 93.2% in each group, respectively, had maintained clinical remission (were relapse free).

**Conclusions:**

MMX mesalazine 2.4 g/day administered as a single or divided dose demonstrated a good safety profile, was well tolerated and was effective as maintenance treatment. High clinical and endoscopic remission rates can be achieved with once-daily dosing.

**Trial registration number:**

NCT00151944.

Mesalazine (5-aminosalicylic acid; 5-ASA), is the standard therapy for maintaining remission in ulcerative colitis (UC).^1^ ^2^ Although currently available mesalazine preparations have been shown to maintain remission, many patients are poorly compliant.^3^^–^7 Patients with quiescent UC who are non-compliant with maintenance 5-ASA therapy have been shown to have a fivefold greater risk of disease flare-ups than compliant patients.^3^ Increased disease activity impacts on patient health and quality of life, may have economic consequences through loss of productivity or earnings, and results in increased hospitalisations, doctors visits and drug costs.^8^^–^13 In addition, regular 5-ASA use may reduce the risk of developing colorectal cancer,^14^ either through decreased disease activity^15^ ^16^ or through a direct anticarcinogenic effect.^17^

As compliance is such a major factor in disease control,^4^ ^6^ ^7^ ^18^ it is important to understand what drives non-adherence and what patients want from their medication. In a recently published internet-based survey of 1595 patients with UC receiving 5-ASA therapy, reasons for poor compliance included forgetting to take medication (stated by >90% of patients), “too many pills”, “dosing required too many times each day”, “medication too inconvenient” and “no symptoms present”,^19^ confirming the results of previous studies.^3^ ^6^ ^20^ ^21^ In the internet survey, patients also expressed a wish for convenient, simple dosing regimens.^19^ Nearly a quarter (23%) of the patients surveyed considered fewer pills and less frequent dosing as “very important” attributes when selecting a treatment for their disease, and more than one-third (34%) considered convenience to be very important. A mesalazine preparation that involves the ingestion of fewer tablets, less often, might therefore be considered likely to impact favourably on compliance with maintenance therapy.

In early 2007, mesalazine with MMX Multi Matrix System (MMX) technology (Lialda (Shire Pharmaceuticals Inc., Wayne, Pennsylvania, USA), hereafter referred to as MMX mesalazine) was approved in the USA for the induction of remission of mild to moderate UC in a once-daily oral dose. In Europe, MMX mesalazine (Mezavant XL in the UK; Mezavant elsewhere in the EU) has been approved for the induction and maintenance of clinical and endoscopic remission in patients with active, mild to moderate UC. This high-strength formulation of 5-ASA (1.2 g tablet) utilises MMX technology comprising lipophilic and hydrophilic excipients enclosed within a gastro-resistant, pH-dependent coating.^22^ ^23^ The gastro-resistant film, covering the tablet core, delays the initial release of 5-ASA until the tablet is exposed to pH 7 or higher, normally in the terminal ileum. As the gastro-resistant coating disintegrates, it is thought that intestinal fluids interact with the hydrophilic excipient causing the tablet to swell (much like a sponge in water) and form an outer viscous gel mass. The viscous gel mass is believed to slow diffusion of the 5-ASA from the tablet core into the colonic lumen. As the tablet core and its surrounding gel mass progress through the colon, it is thought that pieces of the gel mass gradually break away from the core, releasing 5-ASA. It is supposed that the lipophilic excipient slows the penetration of aqueous fluids into the tablet core, reducing the rate of dissolution and thus prolonging therapeutic activity. The combination of the high dose of 5-ASA per tablet coupled with the MMX drug delivery technology allows an effective dose of 5-ASA to be delivered throughout the colon in a single daily dose.

In two previous phase III, randomised, placebo-controlled studies, by Lichtenstein *et al*^24^ (SPD476-301) and Kamm *et al*^25^ (SPD476-302), MMX mesalazine given as 2.4 g once daily, 1.2 g twice daily or 4.8 g once daily was shown to be effective for the induction of clinical and endoscopic remission in patients with active, mild to moderate UC. To date, no data regarding the long-term safety or efficacy of MMX mesalazine, or any other oral 5-ASA, given once daily, have been published as a full article. This study (SPD476-303 (clinical trial registry web address as of 21 July 2007: http://www.clinicaltrial.gov/ct/show/NCT00151944?order = 1)) aimed to investigate and compare the long-term safety and efficacy of maintenance MMX mesalazine 2.4 g/day, given either as a once-daily or a twice-daily divided dose in patients with UC in remission.

## METHODS

### Patients

Male and female patients were entered into this maintenance study following the induction of remission after an acute flare of mild to moderate UC. Patients were enrolled directly following up to 8 weeks’ treatment for acute disease, in the studies reported by Lichtenstein *et al* and Kamm *et al*,^24^ ^25^ hereafter referred to as the “parent studies”, or following a further 8-week extension, study 303. Patients who achieved clinical and endoscopic remission (defined as a modified UC Disease Activity Index (UC-DAI) score of ⩽1, with rectal bleeding and stool frequency scores of 0; a combined Physician’s Global Assessment (PGA) and sigmoidoscopy score of ⩽1, no mucosal friability and an additional requirement for a ⩾1-point reduction from baseline in sigmoidoscopy score) during the parent studies could directly enter into the 12-month, randomised maintenance phase of this study. Patients not in remission by the end of the parent studies, and those who withdrew early after week 2, could enter an 8-week extension phase of study 303 and receive open-label MMX mesalazine 4.8 g/day (2.4 g given twice daily) for 8 weeks. Patients in remission at the end of this 8-week extension phase were then eligible to enter the randomised maintenance phase.

Although not defined in the protocol, some additional patients who were not in strictly defined remission (as above), but deemed by their doctor to be well enough at the end of the parent studies or the 8-week extension phase, could enter the randomised maintenance phase of study 303.

All patients were required to have a satisfactory medical assessment, with no clinically relevant abnormality other than UC. Patients withdrawn from the parent studies, because of a severe or serious adverse event (SAE), were not eligible. Co-administration of corticosteroids (systemic or rectal), other formulations containing 5-ASA, or immunosuppressants were not permitted.

The study was conducted in accordance with current applicable regulations and Good Clinical Practice (GCP) guidelines, and complied with the principles of the amended Declaration of Helsinki. The institutional review board or ethics committee at each site approved the protocol and subsequent amendments. All patients gave written, informed consent.

Here we describe the study design and results for the 12-month, randomised maintenance phase.

### Study design

**Figure 1:**
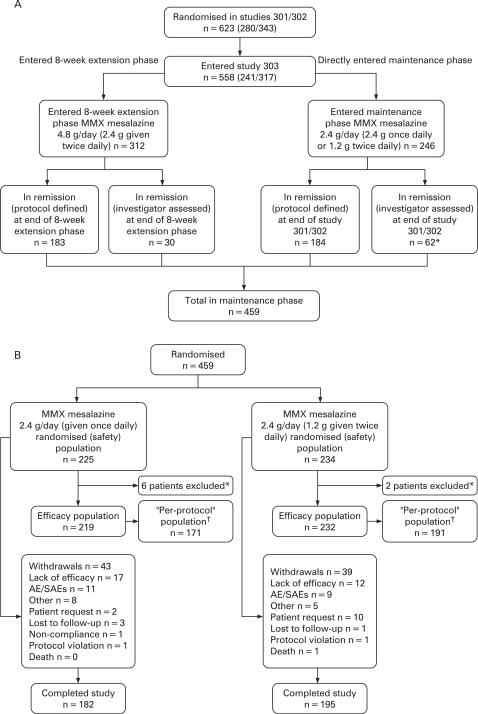
(A) Overall study design (safety population) and (B) patient flow in the 12-month, randomised, maintenance phase of study SPD-476-303. *Patients excluded from the efficacy population because of study centre Good Clinical Practice non-compliance. ^†^The “per-protocol” population included only those patients in the efficacy population who met the strict protocol-defined criteria for remission. AEs, adverse events; SAEs, serious adverse events.

Patients entering this 12-month maintenance study were randomised via an interactive voice recognition system to unblinded therapy with either MMX mesalazine 2.4 g/day (given once daily) or MMX mesalazine 2.4 g/day (1.2 g given twice daily). Patients entered through the two parent studies, which recruited patients from 101 centres across 19 countries (Australia (n = 3), the Czech Republic (n = 16), Estonia (n = 10), France (n = 3), Germany (n = 10), Hungary (n = 31), India (n = 71), Israel (n = 14), Latvia (n = 9), Lithuania (n = 14), Mexico, including Costa Rica (n = 18), New Zealand (n = 12), Poland (n = 132), Romania (n = 11), Russia (n = 113), Spain (n = 7), the Ukraine (n = 78) and the USA (n = 71)). The enrolment and treatment of patients during the current study are summarised in fig 1.

Patients visited the clinic at month 0 (this visit was the same as the end-of-study visit of the parent studies, or the end-of-study visit of the 8-week extension phase of study 303), and then at months 1, 3, 6, 9 and 12. At month 0, patients had: a physical examination; haematology, biochemistry and urine evaluation; sigmoidoscopy, symptoms assessment, PGA, drug compliance check (by pill count), adverse event (AE) review and concomitant medication review. Vital signs were also recorded and, if applicable, a pregnancy test was performed. Patients reported rectal bleeding and stool frequency symptoms (as outlined by the modified UC-DAI) for the last available 3 days prior to the visit. Data older than 5 days were not used. Sigmoidoscopy was performed and inflammation scored in the worst inflamed area in the rectum, or in the sigmoid colon if the rectum was not inflamed.

During the clinic visits at months 1, 3, 6, 9 and 12 (or at the early withdrawal visit), all of the above assessments were carried out, excluding sigmoidoscopy and PGA, which were only performed at the final study visit.

### Primary objectives and outcomes

Given the lack of previous safety data for once-daily maintenance mesalazine therapy, including MMX mesalazine, the pre-defined primary objective of this study was to assess the safety and tolerability of the two dosage regimens over 12 months, including AEs, treatment exposure and time to withdrawal.

All AEs were considered to be “treatment-emergent”, as all patients were being actively treated in the study. AEs were defined as any untoward medical occurrence in a clinical investigation subject who was administered a pharmaceutical product and which did not necessarily have a causal relationship with this treatment. It could, therefore, be any unfavourable and unintended sign (including an abnormal laboratory finding), symptom, disease or exacerbation of the pre-existing condition temporally associated with the use of the medication.

In addition, AEs could be defined as “treatment-related” and were defined as either (1) possibly related to study drug (ie, there may have been some temporal relationship between the event and the administration of the investigational product but there remained some ambiguity as to the cause) or (2) probably related to study drug (ie, the temporal relationship between the event and the administration of the investigational product was compelling, and/or followed a known or suspected response pattern to that product, and the event could not be explained by the subject’s medical condition, other therapies or accident).

All AEs were also classified according to severity:

- Mild: the AE was easily tolerated and did not interfere with usual activity
- Moderate: the AE interfered with daily activity but the subject was still able to function
- Severe: the AE was incapacitating and the subject was unable to work or complete usual activity.

SAEs were defined as any untoward medical occurrence (whether considered to be related to the investigational product or not) that at any dose: resulted in death; was life-threatening (the subject was at risk of death at the time of the event); required inpatient hospitalisation or prolongation of existing hospitalisation; resulted in persistent or significant disability/incapacity; or resulted in a congenital abnormality/birth defect.

### Secondary objectives and outcomes

Predefined secondary outcome measures included comparisons between the two treatment groups for: the proportion of patients in remission (as defined in the parent studies (above)) at 12 months, and the components of the modified UC-DAI score (eg, sigmoidoscopy score).

Assessment of compliance was a predefined analysis whereby patients taking ⩾80% of their prescribed study medication were considered compliant. Compliance with study medication was calculated by pill count.

The UC-DAI (which comprises rectal bleeding, stool frequency, sigmoidoscopy and PGA scores, each assessed on a scale of 0–3 and summed to give a total score of 0–12, as defined by Sutherland *et al* 26) was modified in the parent studies and in this study, so that patients who presented with mucosal friability were given a score of 2 rather than 1. Using this amended scale, mucosal appearance was graded according to the modified UC-DAI, where a score of 0  =  normal; 1 (mild)  =  erythema, decreased vascular pattern and minimal granularity; 2 (moderate)  =  marked erythema, friability, granularity, absent vascular pattern, bleeding with minimal trauma and no ulcerations; 3 (severe)  =  ulceration and spontaneous bleeding. Thus, patients with any mucosal friability were deemed not to be in remission. The PGA is a doctor-based evaluation that considers the scores for rectal bleeding, stool frequency and sigmoidoscopy together with the patient’s general well-being and abdominal discomfort.

### Study populations

The safety population was defined as all patients who received at least one dose of study medication. The efficacy population (“intention-to-treat”) consisted of all patients who received at least one dose of study medication, other than those patients coming from three study sites who, as previously described,^24^ were excluded due to GCP non-compliance.

A retrospectively defined population, hereafter referred to as the “per-protocol” population, included all patients in the efficacy population who met the strict protocol-defined criteria for remission (ie, excluded those patients who were not in remission as defined by the protocol, but were enrolled in the maintenance phase as they were deemed by their doctor to be well enough to receive maintenance treatment).

### Statistical analyses

This study was not designed as a clinical non-inferiority study with minimally acceptable differences between the two treatment regimens. The sample size was dependent on the number of patients in clinical and endoscopic remission (defined above) at the end of the parent studies, or at the end of the 8 weeks of additional therapy with MMX mesalazine 4.8 g/day. As a result of this design, no sample size calculation was performed. Categorical values were summarised using frequencies and percentages, and all statistical comparisons were considered exploratory.

Relapse was defined as a requirement for alternative treatment for UC, including surgery or an increase in the dose of MMX mesalazine above 2.4 g/day. The proportion of patients who were in remission at month 12 was compared between the two treatment groups using the χ^2^ test. All safety summaries were presented for the safety population. Adverse events were coded using the Medical Dictionary for Regulatory Activities (MeDRA) version 5.1. Time to withdrawal was analysed by Kaplan–Meier methodology and measured from the date of the first dose of study medication to the withdrawal date. Treatment differences were analysed using a log-rank test.

## RESULTS

### Patient flow

The study was conducted between 26 November 2003 and 13 March 2006. A total of 459 patients (246 directly from the parent studies and 213 patients who received an additional 8 weeks of treatment with MMX mesalazine 4.8 g/day) were enrolled and randomised (fig 1A).

All patients were evaluable for safety. Eight patients (6 from the once-daily group and 2 from the twice-daily group) were excluded from the efficacy population because of non-compliance and GCP issues. A further 89 patients were excluded from the “per-protocol” population because they did not meet the strict protocol-defined criteria for remission. Three hundred and sixty-two patients were included in the “per-protocol” population, 171 in the MMX mesalazine once-daily group and 191 in the MMX mesalazine divided-dose group (fig 1B).

Overall, 182 (80.9%) patients in the once-daily group and 195 (83.3%) in the twice-daily group completed the study. The most common reason for discontinuation in both dose groups was lack of efficacy/relapse.

### Patient demographics

Baseline demographic characteristics and UC history data were similar for patients in both dose groups (table 1). There were no clinically relevant differences between the two groups with regard to the frequency and type of concomitant medication taken during the study.

**Table 1:** Demographics and baseline characteristics on entry into the parent studies (safety population)

	MMX mesalazine 2.4 g/day (given once daily) (n = 225)	MMX mesalazine 2.4 g/day (1.2 g given twice daily) (n = 234)
Male n (*%*)	106 (47.1)	114 (48.7)
Mean (SD) age, years	42.4 (12.1)	42.6 (13.2)
Non-/previous smoker, n (%)	213 (94.7)	215 (91.9)
Caucasian, n (*%*)	193 (85.8)	202 (86.3)
Diagnosis, n (%)		
Newly diagnosed	32 (14.2)	34 (14.5)
History of UC	193 (85.8)	200 (85.5)
Mean (SD) time since diagnosis, weeks	244.5 (314.1)	288.4 (338.8)
Relapses in last 2 years, n (%)		
0–2	135 (60.0)	144 (61.5)
3–6	76 (33.8)	82 (35.0)
⩾7	4 (1.8)	5 (2.1)
Missing	10 (4.4)	3 (1.3)
Classification of disease*, n (%)		
Left-sided	175 (77.8)	179 (76.5)
Upper limit in transverse colon	14 (6.2)	14 (6.0)
Pancolitis	36 (16.0)	40 (17.1)
Baseline modified UC-DAI score (at parent study entry), mean (SD)	6.3 (1.5)	6.5 (1.4)
Treatment received in parent studies, n (%)		
Placebo	57 (25.3)	61 (26.1)
MMX mesalazine 2.4 g/day	68 (30.2)	67 (28.6)
MMX mesalazine 4.8 g/day	72 (32.0)	70 (29.9)
Asacol	28 (12.4)	36 (15.4)

*Based on patient disease history.

UC, ulcerative colitis; UC-DAI, UC Disease Activity Index.

### Safety

#### Extent of exposure

There was no difference between the two treatment groups with regard to the mean (SD) duration of exposure to study treatment (47.6 (11.1) weeks in the once-daily group and 47.6 (11.4) weeks in the twice-daily group). Although there was a slight gradual decrease in retention (related to the clinical relapse rate) over the 12-month treatment period, the retention rate was in excess of 90% for the first 6 months and was almost 80% for the remainder of the study. The mean (SD) duration of exposure to study treatment was also similar in patients entering directly from the parent studies versus those entering from the 8-week extension phase (48.4 (9.2) weeks vs 46.6 (13.2) weeks, respectively).

#### Treatment-emergent AEs

Overall, 174 patients (37.9%) experienced a total of 384 AEs, the majority of which were mild or moderate in intensity. There were no notable differences between treatment groups with regard to the number and types of AE experienced (table 2). The most frequent AEs were gastrointestinal disorders. Twelve patients (2.6%) had 14 severe AEs. Most severe AEs were gastrointestinal disorders (7 patients (1.5%)), which occurred to a greater extent in the once-daily group (6 patients (2.7%)) than the twice-daily group (1 patient (0.4%)). Only one SAE was considered to be possibly or probably related to study treatment (see below).

**Table 2:** Summary of treatment-emergent and treatment-related adverse events occurring in ⩾2 and ⩾1% of patients, respectively, in any treatment group in the safety population

	Number (%) of patients	
	MMX mesalazine 2.4 g/day (given once daily) (n = 225)	MMX mesalazine 2.4 g/day (1.2 g given twice daily) (n = 234)
Any AE	88 (39.1)	86 (36.8)
Aggravated UC	24 (10.7)	18 (7.7)
Abdominal pain (NOS)	5 (2.2)	4 (1.7)
Abdominal pain upper	1 (0.4)	5 (2.1)
Nasopharyngitis	3 (1.3)	5 (2.1)
Pharyngitis	5 (2.2)	2 (0.9)
Headache	2 (0.9)	5 (2.1)
Any mild AE	62 (27.6)	63 (26.9)
Any moderate AE	44 (19.6)	38 (16.2)
Any severe AE	7 (3.1)	5 (2.1)
Any SAE	9 (4.0)	9 (3.8)
Any AE leading to withdrawal	11 (4.9)	10 (4.3)
Any AE leading to death	0 (0.0)	1 (0.4)
Any treatment-related AE	25 (11.1)	22 (9.4)
Abdominal pain (NOS)	3 (1.3)	2 (0.9)
Colitis ulcerative aggravated	4 (1.8)	1 (0.4)
Diarrhoea (NOS)	3 (1.3)	2 (0.9)
Abdominal pain upper	1 (0.4)	3 (1.3)
Summary of SAEs		
Angina pectoris	0	1
Pulmonary oedema	0	1
UC	5	4
Chronic hepatitis	1	0
Lung abscess	0	1
Pneumonia	0	2
Electric shock	0	1
Abnormal liver function test	1	0
Cerebral infarction	1	0
Aggravated depression	0	1
Menometrorrhagia	1	0
Ovarian cyst	1	0
COPD exacerbation	0	1

AE, adverse event; COPD, chronic obstructive pulmonary disease; NOS, not otherwise specified; SAE, serious adverse event; UC, ulcerative colitis.

#### Treatment-related AEs

Forty-seven patients (10.2%) experienced 76 treatment-related AEs. There were no notable differences between treatment groups with regard to the number and type of treatment-related AEs (table 2). The most frequent treatment-related AEs were gastrointestinal disorders.

#### SAEs

Eighteen patients (3.9%), nine in each group, experienced a total of 22 SAEs (10 in the once-daily group and 12 in the twice-daily group) (table 2). Most SAEs were gastrointestinal disorders, which were experienced by 5 (2.2%) patients in the once-daily group and 4 (1.7%) in the twice-daily group. One SAE (abnormal liver function tests) in the once-daily group was assessed as possibly related to treatment, leading to the patient being withdrawn from the study at the month 1 visit. This patient was noted to have elevated alkaline phosphatase (ALP; 138 U/l; normal range 31–121 U/l), aspartate transaminase (AST; 561 U/l; normal range 1–32 U/l) and alanine aminotransferase (ALT; 1058 U/l) values at month 1. Viral screening revealed a positive latex test for infectious mononucleosis.

#### Discontinuations due to AEs

Twenty-one patients (4.6%) experienced 23 AEs that led to withdrawal (11 patients (4.9%) in the once-daily group and 10 patients (4.3%) in the twice-daily group). Most AEs that led to withdrawal were gastrointestinal disorders (9 (4.0%) patients in the once-daily group and 6 (2.6%) in the twice-daily group). Eleven patients were withdrawn due to SAEs (5 patients in the once-daily group and 6 in the twice-daily group). One patient died due to electric shock.

#### Time to withdrawal

There was no significant difference (p = 0.62) between the two groups in relation to the time to withdrawal, with a small and gradual decrease in retention rate being observed over the 12-month treatment period. The retention rate was in excess of 90% for the first 6 months of the maintenance phase and was in excess of 80% for the remainder of the study.

#### Other safety parameters

There were no remarkable changes in vital signs or clinical laboratory parameters in either treatment group.

### Remission

#### Overall remission

**Figure 2:**
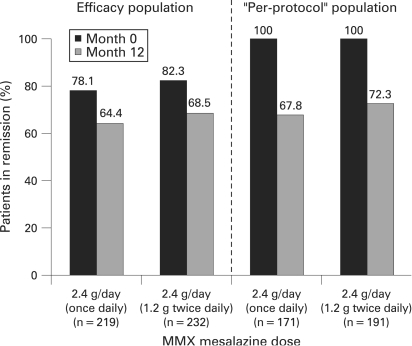
Remission rates at month 0 and month 12 in the efficacy and “per-protocol” populations following treatment with MMX mesalazine 2.4 g/day given once daily or twice daily.

In the efficacy population, 78.1% of patients in the once-daily group and 82.3% of patients in the divided-dose group were in clinical and endoscopic remission at entry (month 0) according to the strict criteria employed in this study (fig 2). At month 12, 64.4% of patients in the once-daily group and 68.5% of patients in the divided-dose group were in strictly defined clinical and endoscopic remission (fig 2). There was no significant difference between the two treatment groups (p = 0.351, odds ratio (OR) 0.83 (95% CI 0.56 to 1.23)).

In the “per-protocol” population in which, by definition, 100% of patients in both groups met the strict remission criteria at month 0, endoscopic and clinical remission were maintained at month 12 in 67.8% of patients in the once-daily group and 72.3% of patients in the divided-dose group (p = 0.359, OR 0.81 (95% CI 0.52 to 1.27)).

#### Remission by entry route and previous treatment in the parent studies

In the efficacy population, of those patients who entered the maintenance phase directly via the parent studies, 75.8% were in remission at month 12 compared with 55.9% of patients who entered via the 8-week extension phase (p<0.0001). Similar results were seen in the “per-protocol” population (80.7% vs 59.7%, respectively; p<0.0001).

**Figure 3:**
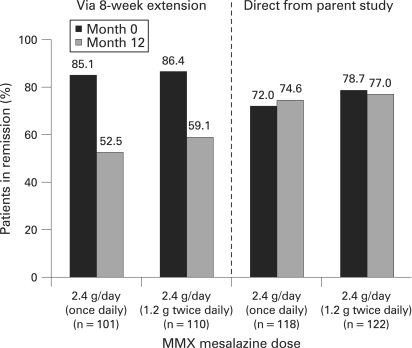
Remission rates at month 0 and month 12 by study entry route (8-week extension phase of study 303 or parent studies) in the efficacy population following treatment with MMX mesalazine 2.4 g/day given once daily or twice daily.

In the efficacy population, remission rates for both dosing regimens were similar for patients entering via the parent studies (74.6% once daily vs 77.0% twice daily, p = 0.655, OR 0.87 (95% CI 0.48 to 1.58)) or via the 8-week extension study, (52.5% once daily vs 59.1% twice daily, p = 0.334, fig 3). Similarly, in the “per-protocol” population, remission rates for both dosing regimens were similar for patients entering via the parent studies (81.2% once daily vs 80.2% twice daily; p = 0.869) or via the 8-week extension phase (54.7% once daily vs 64.2% twice daily; p = 0.190).

Patients’ remission rates, stratified by the treatment received in the parent studies, are shown in table 3.

**Table 3:** Summary of patients in remission at month 12 stratified by previous treatment and entry route (efficacy population; n = 451)

Treatment in parent studies	Entry route into maintenance phase	Patients in remission at the end of 12 months, n (%)		
		MMX mesalazine 2.4 g/day		Total
		2.4 g given once daily (n = 225)	1.2 g given twice daily (n = 234)	
Placebo	Direct	18/22 (81.8)	16/20 (80.0)	34/42 (81.0)
	Via 8-week extension	19/34 (55.9)	25/41 (61.0)	44/75 (58.7)
MMX mesalazine 2.4 g/day*	Direct	25/37 (67.6)	33/42 (78.6)	58/79 (73.4)
	Via 8-week extension	14/29 (48.3)	13/24 (54.2)	27/53 (50.9)
MMX mesalamine 4.8 g/day	Direct	30/42 (71.4)	32/41 (78.0)	62/83 (74.7)
	Via 8-week extension	15/27 (55.6)	17/28 (60.7)	32/55 (58.2)
Asacol 2.4 g/day†	Direct	15/17 (88.2)	13/19 (68.4)	28/36 (77.8)
	Via 8-week extension	5/11 (45.5)	10/17 (58.8)	15/28 (53.6)

*Patients received MMX mesalazine 2.4 g/day given once daily in the Kamm study,^25^ and as 1.2 g twice daily in the Lichtenstein study.^24^ †Patients received Asacol 2.4 g/day (given as 0.8 g three times daily) as a reference arm in study 302 only.

#### Remission rates for patients who did not meet strict entry criteria at baseline (n = 89)

Eighty-nine patients entered the maintenance phase of this study who were not in strictly defined clinical and endoscopic remission at baseline, but who were considered to be well enough by their treating doctor. Their remission rates were less than those achieved by the intention-to-treat population as a whole. Of these 89 patients, remission rates at 12 months were similar between patients dosed once daily (52.1% (25/48 patients)) or twice daily (51.2% (21/41 patients) p = 0.935).

Of these patients, those who entered the maintenance phase through the 8-week extension phase, there was no significant difference between the two treatment groups in relation to endoscopic and clinical remission (40% once daily (6/15 patients) vs 26.7% twice daily (4/15 patients) p = 0.44). Similar numbers of patients who entered the maintenance phase directly from the parent study were in remission at 12 months irrespective of maintenance therapy dose regimen (57.6% once daily (19/33 patients) vs 65.4% twice daily (17/26 patients) p = 0.541).

### Relapse rates

At 12 months, the proportion of patients in the efficacy population who had not relapsed was 88.9% in the once-daily group and 93.2% in the twice-daily group. Similarly, in the “per-protocol” population, the proportion of patients who had not relapsed at 12 months was 88.7% in the once-daily group and 92.5% in the twice-daily group.

### Mucosal appearance

The degree of mucosal inflammation at parent study baseline, upon entry to the maintenance phase and at 12 months is shown in table 4 for the efficacy and “per-protocol” populations. In both populations, the majority of patients in both treatment groups had a moderately inflamed mucosal appearance (median sigmoidoscopy score of 2) at parent study baseline. At entry to the maintenance study, the majority of patients in both groups had a normal mucosal appearance (sigmoidoscopy score of 0), with the remainder having a sigmoidoscopy score of 1. At month 12, these sigmoidoscopy scores had largely been maintained; in the efficacy population, approximately 78% of patients had a sigmoidoscopy score of 0 or 1, while in the “per-protocol” population, approximately 81% of patients had a sigmoidoscopy score of 0 or 1. For both the efficacy and “per-protocol” populations, there were no apparent differences between the two dosing regimens in the distribution of sigmoidoscopy scores after 12 months’ therapy.

**Table 4:** Sigmoidoscopy score distribution at parent study baseline, upon entry to the maintenance phase (month 0) and at 12 months

Sigmoidoscopy score	MMX mesalazine 2.4 g/day (given once daily)		MMX mesalazine 2.4 g/day (1.2 g given twice daily)	
	Efficacy population n = 219 (% patients)	“Per-protocol” population n = 171 (% patients)	Efficacy population n = 232 (% patients)	“Per-protocol” population n = 191 (% patients)
Parent study baseline				
0 (normal)	0	0	0	0
1 (mild)	18.7	18.1	12.5	11.5
2 (moderate)	74.9	77.2	80.6	81.7
3 (severe)	6.4	4.7	6.9	6.8
Month 0				
0 (normal)	66.2	66.1	58.6	59.7
1 (mild)	33.8	33.9	41.4	40.3
2 (moderate)	0	0	0	0
3 (severe)	0	0	0	0
Month 12				
0 (normal)	57.1	58.5	56.9	59.7
1 (mild)	21.5	22.2	21.6	20.9
2 (moderate)	3.2	2.9	3.4	2.6
3 (severe)	0.5	0	1.7	2.1
missing	17.8	16.4	16.4	14.7

### Compliance

No notable differences in compliance rates were observed between treatment groups (safety population) at any visit. Overall, 442 (96.3%) patients took ⩾80% of their prescribed study medication (93.3% in the once-daily group and 99.6% in the twice-daily group).

## DISCUSSION

This study is the first to describe the safety and efficacy of MMX mesalazine administered once or twice daily for the maintenance of remission of UC, over a period of 12 months. To our knowledge, to date no data have been published in a full article regarding the long-term safety of once-daily 5-ASA use or long-term use of MMX technology. MMX mesalazine 2.4 g/day (given once daily or 1.2 g twice daily) was shown to be well tolerated over the 12-month period. The withdrawal rate was very low, with a >80% retention rate at 12 months. During 12 months of treatment, AEs and treatment-related AEs were infrequent, generally mild to moderate in intensity, and gastrointestinal in nature (most commonly related to the underlying condition). Severe AEs, most commonly gastrointestinal events, were also rare. While a greater number of severe AEs were reported in the once-daily group, no severe AE in either dose group was considered to be related to study medication. Only one SAE (abnormal liver function tests) was considered possibly related to treatment. Although the current study did not include a placebo arm, the AE profile reported here is similar to those reported in long-term studies of other non-sulphur-containing 5-ASA formulations, where AEs were also infrequently reported, usually of mild to moderate severity and mainly gastrointestinal in nature.^27^^–^36 The safety results of the present study also support the results of a recent meta-analysis of prospective, randomised, double-blind, and controlled trials of newer release 5-ASA formulations showing that long-term 5-ASA use incurs no more AEs than placebo.^37^ Pancreatitis, a known idiosyncratic side effect of mesalazine, was seen in one of the parent studies,^24^ but not seen in this maintenance study, presumably because patients were stable on the medication and because of its rarity.

Of equal importance to safety, the long-term efficacy of once-daily maintenance therapy has not been published. In this study, the pre-specified end points included the long-term efficacy. MMX mesalazine maintained clinical and endoscopic remission over the 12-month study period in 64.4 and 68.5% in the once-daily and divided-dose groups, respectively. This remission rate refers to those patients who had experienced no flare-up during the study and fulfilled the other strict remission criteria, including endoscopic remission.

Approximately 20% of patients who entered into the maintenance study were not in remission (as defined by the strict criteria in the protocol) at month 0, but were deemed well enough by individual investigators to receive maintenance therapy. When these patients were excluded from the analyses, strictly defined clinical and endoscopic remission rates of approximately 70% were observed at month 12.

The remission rates for those not in full endoscopic and clinical remission were less than those achieved by the intention-to-treat populations as a whole, suggesting that full endoscopic and clinical remission at entry were more likely to result in maintained remission at 12 months.

As well as maintaining strict clinical and endoscopic remission, very few patients relapsed while receiving MMX mesalazine—that is, 12% of patients in the once-daily group and 8% of patients in the twice-daily group (all evaluable patients). There was also no significant difference between the two dosing regimens with regards to time to relapse. The study population comprised patients who had experienced a disease flare-up immediately prior to entering the maintenance study (ie, were part of the active disease parent studies). Such a population might be expected to contain more patients who will subsequently relapse than a study population consisting of patients who have been in remission for a long time.^38^

The design of the trials with MMX measalazine has allowed investigation of the same study population through both induction and subsequent maintenance of UC remission with MMX mesalazine. The study population enrolled in this 303 maintenance trial was therefore characterised by a good response to mesalazine in the parent induction of remission trials. These patients may be more mesalazine responsive than the total population of colitic patients. The results presented here have shown that patients receiving MMX mesalazine and patients who received Asacol (internal reference arm of the Kamm study^25^) during the parent studies had similar 12-month remission rates following maintenance therapy with MMX mesalazine. Therefore, MMX mesalazine appears to be efficacious as maintenance therapy regardless of the 5-ASA formulation used to induce remission.

Overall, 5-ASA formulations have been shown to be generally effective for maintenance of UC remission. In a meta-analysis of oral 5-ASA formulations for the maintenance of remission of UC, the Peto OR for the failure to maintain clinical or endoscopic remission for oral 5-ASA versus placebo was 0.47 (95% CI, 0.36 to 0.62).^37^ However, long-term (>6 months) remission rates, and clinical and endoscopic response rates, reported in previous studies vary considerably. For example, in a 12-month, randomised, follow-up study of 80 patients with quiescent UC receiving oral mesalazine 2.4 g/day, only 30% of patients were in remission at the end of the study.^39^ In contrast, a double-blind, randomised study comparing oral controlled-release mesalazine 4 g/day (Pentasa^®^; available from Shire Pharmaceuticals, Wayne, Pennsylvania, USA) with placebo demonstrated that after 12 months, 64% and 38% of patients, respectively, had not relapsed (defined by endoscopic appearance, stool frequency and the presence of rectal bleeding), and thus were considered to be in remission.^27^

The variability in reported remission rates is, in part, due to the diverse range of clinical and endoscopic indices utilised by the investigators in such studies, and the subsequent variability in the definition of remission. For instance, the recent PODIUM (Pentasa Once Daily In Ulcerative colitis for Maintenance of remission) trial defined remission as a UC-DAI score of <2.^40^ In the present study remission was defined as a UC-DAI score of ⩽1. In the original UC-DAI, mucosal friability is allowed at a score of 1; however, for additional stringency, we excluded any patient with mild mucosal friability on endoscopy. This is important as recently published data suggest that those patients who achieve mucosal healing are less likely to relapse than those who do not.^41^ Moreover, the presence of macroscopic large bowel inflammation has also been shown to be an important independent determinant of the risk of colorectal neoplasia in patients with long-standing UC.^15^ It is clear that the clinical relevance of trial end points should be considered when validating claims regarding efficacy and dosing regimens. Direct comparisons of oral 5-ASA formulations dosed once daily for the maintenance of UC are required.

Results obtained from the ITT population and the “per-protocol” populations were similar, indicating that patients who did not fully achieve remission during the parent trials could still achieve and maintain clinical and endoscopic remission when receiving MMX mesalazine as maintenance therapy at a dose of 2.4 g/day. This is a reassuring result, as in clinical practice a doctor may consider a patient to be in remission without performing an endoscopy. Remission rates at 12 months with MMX mesalazine were higher in patients who entered the maintenance study directly from the parent studies compared with those who entered via the 8-week extension phase of study 303. This suggests that patients able to achieve remission with MMX mesalazine during an 8-week period may represent a more mesalazine-responsive patient population, relative to those who required an additional 8 weeks of treatment. Moreover, our findings suggest that patients who are more quickly responsive during therapy for achieving remission may be more likely to be maintained in remission during MMX mesalazine maintenance therapy.

Maintenance therapy of remission with MMX mesalazine was associated with favourable endoscopic findings, such that, at month 12, the majority of patients in both treatment groups (57%) had a normal mucosal appearance. The vast majority of the remaining patients with available severity data (>80%), in either treatment group, had a sigmoidoscopy score of only 1 at month 12, and only a very small percentage of patients (<1.5%) had a worsened mucosal appearance from parent study baseline. Very similar observations were made in the “per-protocol” population.

Despite the long duration of the study, 93.3% of patients in the once-daily group and 99.6% of patients in the twice-daily group were ⩾80% compliant with the study medication. While the compliance rate was extremely high, it was not unexpected given the fact that patients were closely monitored in a clinical trial environment, where compliance rates are traditionally high. However, the disparity between compliance rates in controlled trials and the community setting is well known.^20^ It remains to be seen if the compliance rates achieved in the present study will translate into improved compliance in general clinical practice compared with the standard multiple daily dose, high tablet burden, 5-ASA regimens currently in use.

The current study did not include a placebo arm. When designing the study such a placebo comparison was considered unethical, given the established efficacy and low toxicity of 5-ASA therapy in maintaining remission. The high clinical remission (relapse free) rate seen in this study is substantially greater than seen in previous early placebo-controlled and observational studies.

In summary, once-daily MMX mesalazine appears to have a similar safety and efficacy profile to twice-daily MMX mesalazine, in the maintenance of remission of ulcerative colitis.
